# Transposition of left subclavian artery with reimplantation of isolated left vertebral artery before thoracic endovascular aneurysm repair for type B aortic dissection

**DOI:** 10.1016/j.jvscit.2022.03.004

**Published:** 2022-03-22

**Authors:** Michael Chaney, Victor Martinez-Zavala, Rym El Khoury, Gaurang Joshi, Chad E. Jacobs, John V. White, Lewis B. Schwartz

**Affiliations:** a Department of Surgery, Advocate Lutheran General Hospital, Park Ridge, IL; b Division of Vascular Surgery, University of California, San Francisco, San Francisco, CA

**Keywords:** Aberrant anatomy, Aortic dissection, TEVAR, Transposition

## Abstract

Understanding and recognizing anatomic anomalies of the aortic arch is important when planning extra-anatomic debranching before thoracic endovascular aortic repair. A rare anomaly is the left vertebral artery aberrantly arising from the aortic arch; found in ∼5% of adults. When present, the artery courses through the carotid sheath at a variable length before entering the third or fourth cervical transverse foramen. In the present report, we have described the case of a 49-year-old man with a symptomatic, enlarging type B aortic dissection with an aberrant left vertebral artery and the novel methods used to surgically correct his pathology.

Acute type B aortic dissection (TBAD) is a rare, but life-threatening, condition with an annual incidence of 3.5/100,000 persons.^1^ Thoracic endovascular aortic repair (TEVAR) has become the standard of care for this complex disease process.^2^^,^^3^ Although emergency repair is indicated for TBAD acutely complicated by malperfusion or rupture, the treatment of uncomplicated cases has remained primarily medical, with a focus on anti-impulse therapy with strict blood pressure and heart rate goals. For these patients, frequent repeat imaging is key to identifying disease progression, which can include distal or proximal extension and/or aneurysmal degeneration, because ≤45% of patients presenting with acute TBAD will develop aortic enlargement within the first year and 20% will eventually require intervention.^4^^,^^5^

The treatment of choice for TBAD has become TEVAR. In the setting of aneurysmal degeneration, the primary goals include coverage of the entry tear and exclusion of the aortic aneurysm. In cases in which the intimal tear is in close proximity to the origins of the great vessels, adjunctive bypass or transposition (“debranching”) could be necessary to minimize the risk of cerebral, spinal cord, and upper extremity ischemia.^6^ High-quality, preprocedure imaging is essential for operative planning and to identify any aortic arch anomalies, which occur frequently in aortic dissection that includes a common origin of the innominate and left common carotid arteries (bovine arch, ∼30%) and an isolated left vertebral artery arising from the arch (∼3%).^7^, ^8^, ^9^ In the present report, we have described the clinical course of a patient with TBAD and the latter anomaly: an aberrant left vertebral artery originating directly from the aortic arch. He was successfully treated with staged surgical left carotid–subclavian transposition with reimplantation of the left vertebral artery into the transposed left subclavian artery, followed by TEVAR.

## Case report

The patient was a 49-year-old man with hypertension, hyperlipidemia, and chronic kidney disease who had presented as the driver in a motor vehicle accident during which he had lost consciousness. On extrication, he was noted to have left hemiplegia highly suspicious for stroke. On arrival to the hospital, his blood pressure was 239/137 mm Hg. Right facial droop and left hemiplegia were present. Pulses were palpable in both wrists and feet. The patient was in respiratory distress and thus was intubated and sedated. The patient’s wife, who had power of attorney for personal care, and the patient both provided verbal consent for the report of his case details and imaging studies.

**Fig 1:**
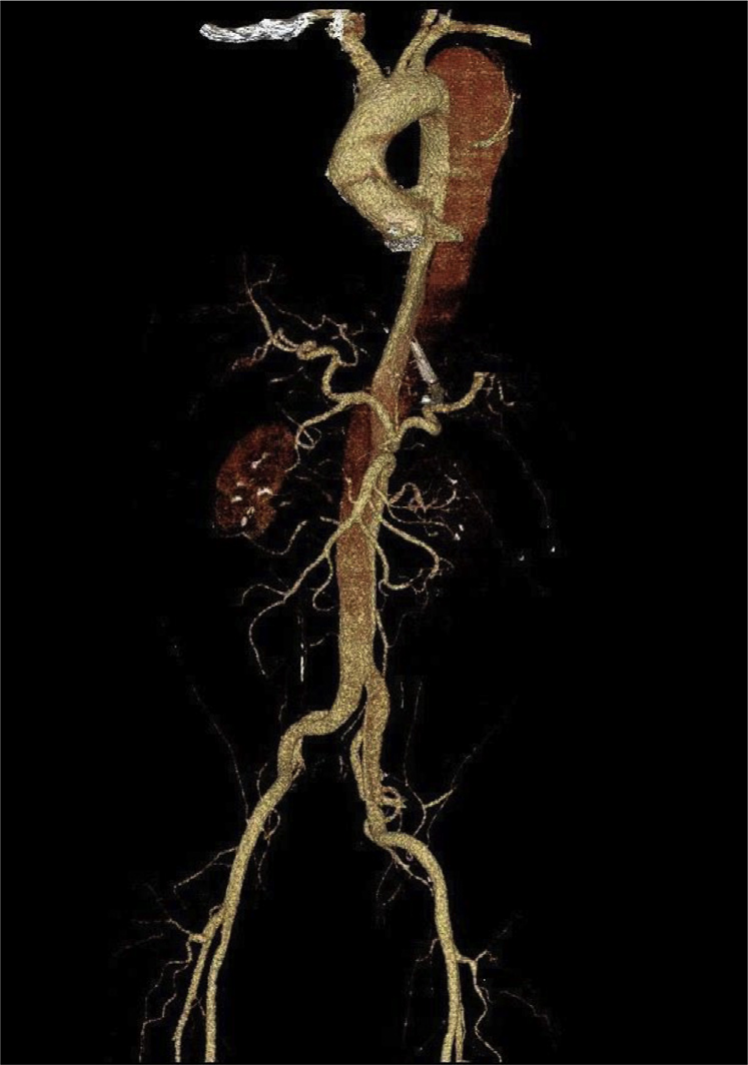
Computed tomography (CT) scan of the chest of 49-year-old man revealing an extensive type B aortic dissection (TBAD) originating in the proximal descending thoracic aorta just distal to the takeoff of the left subclavian artery. The dissection extended to involve the entirety of the abdominal aorta and both common iliac arteries. The true lumen supplied the celiac and mesenteric arteries and the left renal artery. The false lumen supplied the right renal artery. The dissection was graded as type B, 2, 10.^8^ For Type B dissections gradings can include the proximal (2 which includes the arch portion with the left subclavian origin) and distal (10 into the common illiacs) limits of the false lumen.^28^

Computed tomography (CT) of the head revealed an acute 5.2 × 2.9 × 6.0-cm, right hemispheric intraparenchymal hematoma centered within the putamen most consistent with hypertensive hemorrhage. Intraventricular extension with mild temporal horn dilatation and a 3-mm midline shift to the left was found. CT of the chest revealed an extensive TBAD originating in the proximal descending thoracic aorta just distal to the takeoff of the left subclavian artery (Fig 1). The dissection extended to involve the entirety of the abdominal aorta and affected both common iliac arteries. The TBAD also involved the proximal left external iliac artery. The true lumen supplied the celiac and mesenteric arteries and the left renal artery. The right renal artery was perfused by the false lumen.

The patient underwent immediate placement of a left-sided external ventricular drainage catheter, followed by craniotomy and minimally invasive evacuation of the large, intracerebral hematoma. His course was complicated by persistent, severe neurologic deficit, poor oxygenation with adult respiratory distress syndrome, anemia, pneumonia, and renal failure. He was treated with antihypertensive agents, hemodialysis, tracheostomy, mechanical ventilation, and enteral nutrition. Repeat cross-sectional imaging was performed on hospital day 3, which revealed the absence of dissection progression or aneurysmal changes. The patient was discharged to a rehabilitation facility with a requirement for hemodialysis after a 2-month hospital stay. He underwent rehabilitation for an additional 1 month and then returned home.

**Fig 2:**
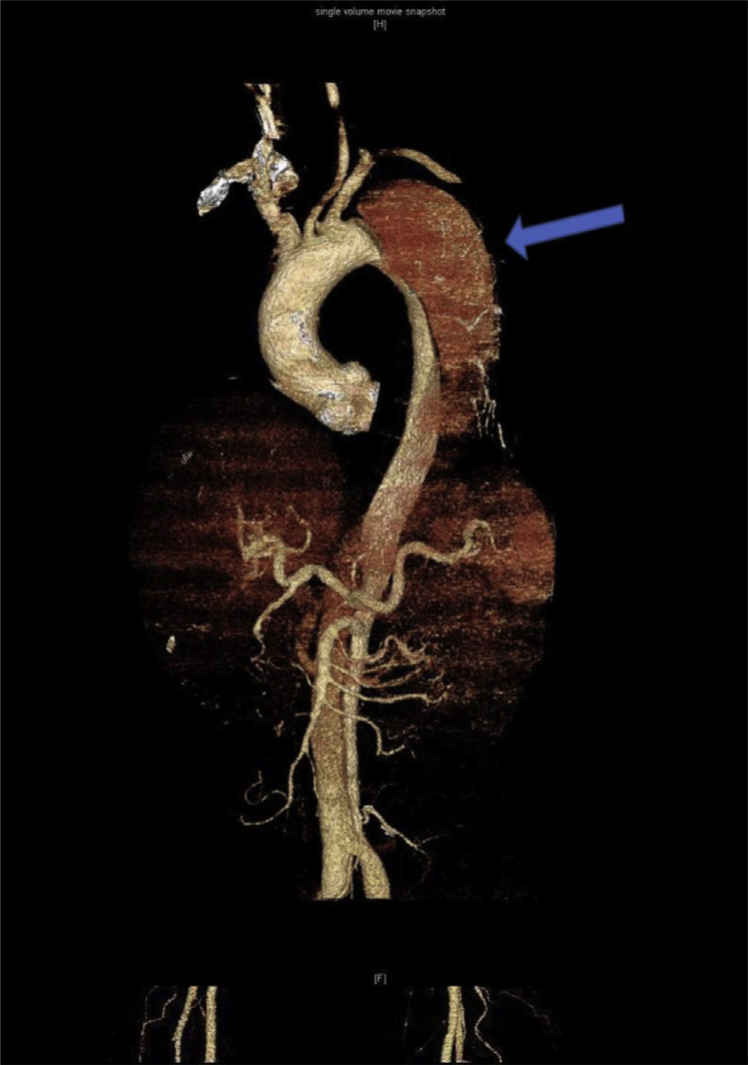
Follow-up computed tomography (CT) scan after 5 months again demonstrating type B aortic dissection (TBAD; *arrow*). The proximal descending aorta measured 6.5 cm (increased from 6.1 cm).

**Fig 3:**
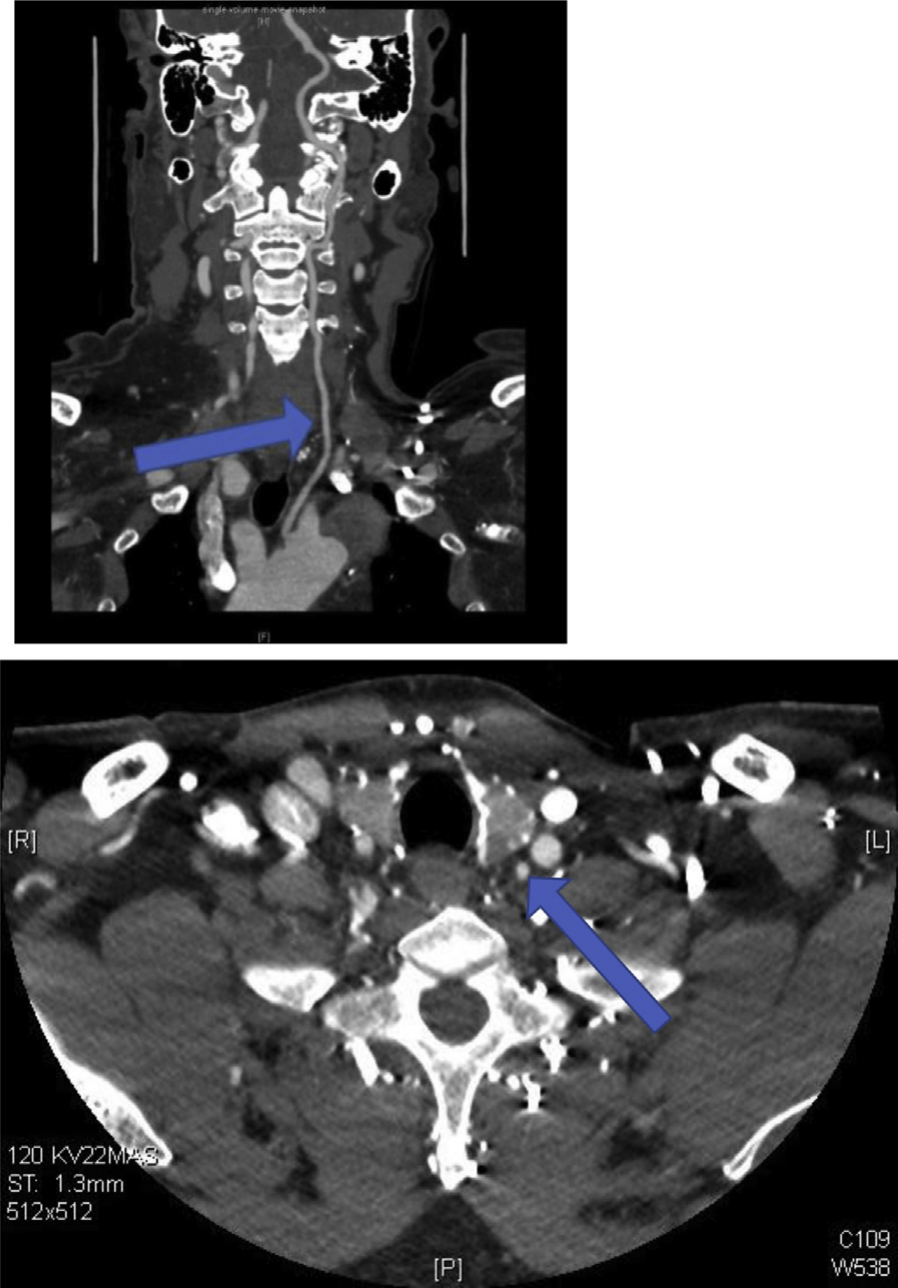
Follow-up computed tomography (CT) scans after 5 months showing left vertebral artery aberrantly arising from the aortic arch (*blue arrow*; **Top**) and the left vertebral artery coursing posteriorly in the left carotid sheath (*blue arrow*) before entering the C6 transverse foramen (**Bottom**).

The patient remained hemiplegic and required continuous hemodialysis but was awake and communicative. A follow-up CT scan at 6 months again demonstrated the TBAD. The maximal diameter of the proximal descending aorta had increased from 6.1 to 6.5 cm. However, the morphology of the TBAD was otherwise unchanged (Fig 2). It was confirmed that the left vertebral artery arose from the aortic arch and coursed posteriorly in the carotid sheath before entering the C6 transverse foramen (Fig 3). His right vertebral artery was dominant and patent. Given the large and increasing size of the false channel, TEVAR was indicated. The left subclavian artery origin was measured at 7 mm from the proximal extent of the dissection. To establish an adequate proximal landing zone, antecedent left subclavian and vertebral artery transposition was required.

The patient was taken to the operating room electively for left carotid–subclavian–vertebral artery transposition under general anesthesia. A 7-cm transverse incision was made just above the left clavicle, and the scalene fat pad was retracted superiorly. The brachial plexus, phrenic nerve, and left subclavian vein were all identified and preserved. The left subclavian artery was identified inferiorly in the wound and was dissected proximally and distally for a distance of 6 cm and encircled with vessel loops. The artery was mobilized in its entire length to facilitate transposition.

The sternocleidomastoid muscle was retracted laterally, and the carotid sheath entered. The internal jugular vein and vagus nerve were identified and preserved. The carotid artery was dissected proximally and distally for a distance of 4 cm. Minimal atherosclerotic changes were observed. A small additional artery, presumed to be the aberrant left vertebral artery, was found posterior to the common carotid artery within the carotid sheath. It was dissected proximally and distally for a distance of 4 cm in preparation for the transposition.

**Fig 4:**
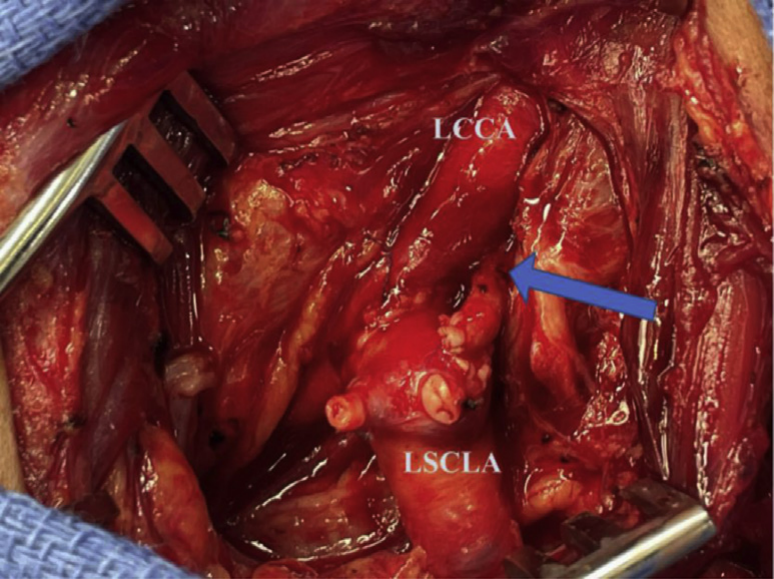
Intraoperative photograph of completed left carotid–subclavian–vertebral transposition. The *blue arrow* indicates the aberrant left vertebral artery transposed on the superior margin of the transposed left subclavian artery (*LSCLA*). *LCCA,* Left common carotid artery.

After administration of heparin, the left subclavian artery was clamped distally, oversewn, and divided proximally. The common carotid artery (proximal to its bifurcation) was clamped proximally and distally with atraumatic vascular clamps, and a longitudinal arteriotomy was made on its anterolateral surface. An end-to-side anastomosis of the divided left subclavian artery to the left common carotid artery was performed. After the carotid–subclavian transposition, the left vertebral artery was clamped and divided inferiorly in the wound adjacent to the proximal common carotid artery. Its proximal stump was ligated. The distal artery was transposed superficially in the wound and anastomosed to the transposed left subclavian artery. After clamp release, strong pulses were noted throughout the vascular reconstruction (Fig 4). The patient’s neurologic status was unchanged postoperatively. The patient recovered uneventfully and was discharged after 2 days without ongoing antithrombotic therapy.

**Fig 5:**
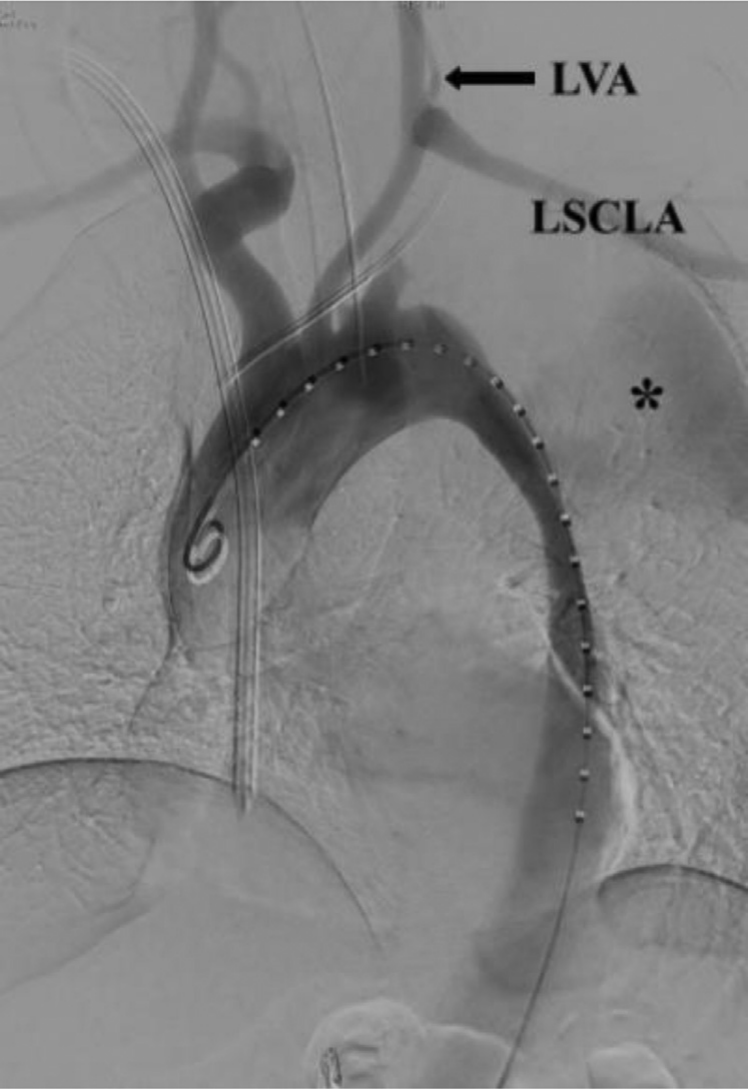
Thoracic aortic aneurysm repair (TEVAR) in a 49-year-old man with type B aortic dissection (TBAD). Note the exclusion of the large, false channel (*asterisk*) after graft implantation and patency of the transposed left subclavian artery (*LSCLA*) and left vertebral artery (*LVA*).

The patient was returned to the operating room 2 weeks later for TEVAR. A prophylactic lumbar drain was placed before the induction of general anesthesia. A 36-mm × 150-mm thoracic stent graft (Captiva; Medtronic, Dublin, Ireland) was advanced and deployed using right femoral access. The transposed left subclavian and left vertebral arteries were widely patent (Fig 5). The procedure was uncomplicated, and the lumbar drain was removed on postoperative day 1. The patient was discharged after 4 days.

Follow-up CT angiogram at 8 months demonstrated a patent TEVAR stent graft without an endoleak, with the aneurysmal segment measuring 7.8 cm × 6.4 cm (previously had measured 8.7 cm × 6.3 cm). The imaging studies also showed the left subclavian artery had become occluded at its origin, with a widely patent left carotid–subclavian–vertebral transposition.

## Discussion

In the present report, we have described the rapid aneurysmal degeneration of a TBAD in a 49-year-old man with a complex presentation, including severe hypertension, trauma, intraparenchymal cerebral hemorrhage, and renal failure. The quick progression of his condition and the unusual course of the left vertebral artery arising directly from the diseased aorta prompted a staged repair, which included carotid–subclavian–vertebral transposition, followed by TEVAR. The patient recovered uneventfully, and his outcome was favorable.

In the present patient, who was already disabled by cerebral infarction from an extensive intraparenchymal hemorrhage, preservation of the systemic left vertebral and left subclavian arterial pressure and flow at TEVAR was believed to be indicated. Although somewhat controversial, the results from a recent meta-analysis have suggested that left subclavian revascularization in adjunct to TEVAR will significantly decrease the risk of spinal cord ischemia, cerebrovascular accident, endoleak, and left upper extremity ischemia.^10^, ^11^, ^12^ For patients with an isolated left vertebral artery, the indication is probably absolute. Autogenous subclavian artery reconstruction, in contrast to prosthetic carotid–subclavian bypass, offered several advantages for our patient, including potentially enhanced primary patency, freedom from recurrent symptoms, and a reduction of infectious complications.^5^^,^^13^^,^^14^

Anomalies of the aortic arch are common, with a normal, three-vessel branching pattern only observed in ∼60% of adults.^15^, ^16^, ^17^ The most common variant is the (unfortunately and incorrectly named) “bovine aortic arch,” with a prevalence as high as 40%.^18^ The second most common anomaly is an “isolated” left vertebral artery, taking its origin from the aortic arch, instead of the left subclavian artery. This has been shown to occur in 0.79% to 8.3% of adults, certainly common enough to be encountered by most vascular surgeons during their career.^9^^,^^19^, ^20^, ^21^, ^22^, ^23^, ^24^ Other aberrant parent vessels of the vertebral arteries have also occasionally been described, including the external carotid artery, thyrocervical trunk, common carotid artery, internal carotid artery, carotid bulb, costocervical trunk, occipital artery, inferior thyroid artery, and ascending aorta.^9^^,^^21^^,^^22^^,^^24^ Aortic arch anomalies have been frequently observed in patients with TBAD, although their prevalence has not been shown to be higher than that in the general population.^7^ Although still uncertain, the results from at least one meta-analysis have positively associated the presence of arch anomalies with aortic pathologies, such as atherosclerosis and dissection.^19^

A normal left vertebral artery will typically arise from the left subclavian artery, course superoposteriorly between the longus colli and anterior scalene muscles, enter the foramen transversarium of the sixth cervical vertebra, and converge in the circle of Willis to supply the spinal cord, brainstem, cerebellum, and occipital lobes.^22^, ^23^, ^24^ However, when originating from the aortic arch, the vessel will typically remain outside the vertebral column for a longer length, coursing anterior and/or lateral to the longus colli muscle until entering the fourth or fifth transverse foramen.^16^^,^^19^^,^^20^^,^^24^^,^^25^ This makes the prevertebral segment of the artery longer (∼8 cm) and more surgically accessible in the neck.^24^ It might even conveniently reside in the posterior aspect of the carotid sheath, such as in the present case. However, in other cases, the vertebral artery aberrancy could complicate vascular reconstruction.^9^

Surgical transposition of an isolated left vertebral artery into a carotid–subclavian bypass graft has been reported in a case of TEVAR for a symptomatic aortic arch aneurysm.^26^ Isolated left vertebral artery transposition to the left common carotid artery has also been performed before TEVAR for intramural hematoma.^27^ The present case is rare owing to use of transposition combined with reimplantation of a vertebral anatomic variant.

## Conclusions

We have reported the uncommon scenario of a patient with chronic TBAD and an aberrant left vertebral artery originating from the arch, who had undergone combined carotid–subclavian–vertebral transposition before successful TEVAR. The possibility of this common aortic arch variant should be considered when planning surgical treatment for TBAD.

Anatomic variations can complicate surgical decision-making for patients with chronic TBAD. Careful preoperative assessment of the aortic arch anatomy, combined with operative strategies to preserve cerebrovascular and vertebral vascular blood flow, are essential to optimizing treatment outcomes.
